# A spatiotemporal analysis of the association between carbon productivity, socioeconomics, medical resources and cardiovascular diseases in southeast rural China

**DOI:** 10.3389/fpubh.2023.1079702

**Published:** 2023-07-06

**Authors:** Xuwei Tang, Zhi-Ying Zhan, Zhixiang Rao, Haiyin Fang, Jian Jiang, Xiangju Hu, Zhijian Hu

**Affiliations:** ^1^Department of Epidemiology and Health Statistics, School of Public Health, Fujian Medical University, Fuzhou, China; ^2^Medical Department of Fujian Provincial Hospital, Fuzhou, China; ^3^Fujian Center for Disease Control and Prevention, Fuzhou, China

**Keywords:** geographically and temporally weighted regression model (GTWR), multiscale geographically weighted regression (MGWR), cardiovascular diseases, rural China, carbon productivity

## Abstract

**Introduction:**

With China’s rapid industrialization and urbanization, China has been increasing its carbon productivity annually. Understanding the association between carbon productivity, socioeconomics, and medical resources with cardiovascular diseases (CVDs) may help reduce CVDs burden. However, relevant studies are limited.

**Objectives:**

The study aimed to describe the temporal and spatial distribution pattern of CVDs hospitalization in southeast rural China and to explore its influencing factors.

**Methods:**

In this study, 1,925,129 hospitalization records of rural residents in southeast China with CVDs were analyzed from the New Rural Cooperative Medical Scheme (NRCMS). The spatial distribution patterns were explored using Global Moran’s I and Local Indicators of Spatial Association (LISA). The relationships with influencing factors were detected using both a geographically and temporally weighted regression model (GTWR) and multiscale geographically weighted regression (MGWR).

**Results:**

In southeast China, rural inpatients with CVDs increased by 95.29% from 2010 to 2016. The main groups affected were elderly and women, with essential hypertension (26.06%), cerebral infarction (17.97%), and chronic ischemic heart disease (13.81%) being the leading CVD subtypes. The results of LISA shows that central and midwestern counties, including Meilie, Sanyuan, Mingxi, Jiangle, and Shaxian, showed a high-high cluster pattern of CVDs hospitalization rates. Negative associations were observed between CVDs hospitalization rates and carbon productivity, and positive associations with *per capita* GDP and hospital beds in most counties (*p* < 0.05). The association between CVDs hospitalization rates and carbon productivity and *per capita* GDP was stronger in central and midwestern counties, while the relationship with hospital bed resources was stronger in northern counties.

**Conclusion:**

Rural hospitalizations for CVDs have increased dramatically, with spatial heterogeneity observed in hospitalization rates. Negative associations were found with carbon productivity, and positive associations with socioeconomic status and medical resources. Based on our findings, we recommend low-carbon development, use of carbon productivity as an environmental health metric, and rational allocation of medical resources in rural China.

## 1. Introduction

Globally, non-communicable chronic (NCDs) diseases have become the leading cause of disease burden. Recently, cardiovascular diseases (CVDs) refer to ischemic or hemorrhagic diseases of the heart, brain, and body caused by factors such as hyperlipidemia, blood viscosity, atherosclerosis, and hypertension, which have the highest mortality rate among NCDs. (1, 2) In China, CVDs were major contributor to the burden of disease, which have caused serious damage to the health of people and the economy. (3) It has previously been noted that the age-standardized prevalence of CVDs in China increased significantly by 14.7% overall, and the number of deaths caused by CVD increased from 2.51 million to 3.97 million, from 1990 to 2016. CVDs account for more than 40% of deaths in China. Compared with cities, the mortality rate in rural areas is higher. In 2016, the mortality rate of CVDs in rural China was 309.33 per 100,000, and 158.15 per 100,000 for cerebrovascular diseases. (4) Most of the risk of CVDs is attributable to relatively few and modifiable risk factors, such as lifestyle, diet, economic status. (5)

Carbon productivity is a composite indicator that takes into account both carbon emissions and economic output. A higher carbon productivity value signifies that more economic output is achieved while emitting less carbon, which is seen as a positive result of reducing the environmental impact of carbon emissions. (6) People’s health could be affected by the carbon productivity of the area where they live, as higher levels of carbon emissions can have negative impacts on human health. Carbon emissions from human activities, such as the burning of fossil fuels, contribute to air pollution, which can have serious health consequences. (7) Numerous studies have shown that air pollution is an independent risk factor for CVDs. (8, 9) The risk of CVDs increases by 0.27% for every 10 mg/m^3^ increase in the average daily PM_2.5_ concentration in China. (10) In addition, the increasing carbon emission has caused the “greenhouse effect” to be responsible for global warming in the past decades. (11) In the context of global warming, cardiovascular hospitalizations will likely increase. (12) Carbon productivity could be a promising metric for evaluating the environmental health of a region. However, there is a scarcity of research on the association between carbon productivity and health outcomes. Further studies are needed to establish a clearer understanding of the association.

Considerable research has examined the relationship between socioeconomics, medical resources and the risk for CVDs from the individuals’ perspective. (13, 14) In rural China, people in lower socioeconomic status groups are more likely to have a higher risk of CVDs (15), which is driven by a range of factors, including education, air pollution, and unhealthy lifestyles. Besides, people from low socioeconomic backgrounds are less likely to have access to adequate medical resources, including preventive services, diagnostic tests, and treatment options, which can help to identify and manage CVDs risk. (16) However, studies of spatial analysis to explore the spatial heterogeneity of these associations are limited, which has advantages for developing regional development plans and efficiently allocating medical resources to maximize benefits for the population.

Recently, geographically weighted regression (GWR) model parameter estimation algorithms have been continuously improved. (17–19) The GWR model provides an intuitive and practical method for spatial analysis of heterogeneity of spatial relationships, which has gradually become an effective approach to exploring the spatial association of disease burden with influencing factors, thus providing a basis for the government to formulate health policies or allocate medical resources rationally. (20–22) Additional studies are needed, as kinds of spatial analysis studies are limited in the field of CVDs.

Fujian is a representative province in the southeastern coastal area of China, comprising 84 counties with a population of about 39 million and an area of 121,400 km^2^. Our previous study found that the number of rural elderly inpatients with CVDs in southeastern China is increasing rapidly, and CVDs were the main cause of catastrophic medical expenses. (23) Thus, this study aimed to further describe the temporal and spatial distribution pattern of CVDs hospitalizations, and to explore the relationship between carbon productivity, socioeconomics, medical resources and hospitalization for CVDs by spatial analysis. The findings are essential for the government to formulate health policies, improve economic development plans, and allocate medical resources rationally to cope with the disease burden of CVDs.

## 2. Materials and methods

### 2.1. Data

#### 2.1.1. Data source

The data of CVDs inpatients for the study were obtained from hospitals in the areas covered by the New Rural Cooperative Medical Scheme (NRCMS) in Fujian Province, China, from 2010 to 2016. To reduce the medical economic burden of rural residents, the Chinese government has guided, organized, and supported NRCMS since 2003, which had achieved generous benefits, and the enrolment rate among rural residents had reached 96.6% in 2010. (24) Accordingly, CVDs hospitalizations among rural residents can be well represented by the NRCMS database. Variables in the NRCMS database include age, gender, NRCMS registration address at the county level, whether they were subsistence allowance households, hospital level (provincial, municipal, county and township level), admission date, and International Classification of Diseases (ICD-10) code. We employed the NCMS registration address, usually the inpatient’s residential address, for analysis, resulting in a limited impact of cross-broader hospitalizations. To protect patient privacy, the data manager removed personally identifiable information before analysis. The causes of hospitalization were classified by the ICD-10. CVDs were defined as follows: hypertension, (I10-I15) heart diseases, (I05-I09, I20-I52) cerebrovascular diseases (I60-I69, G45, G46) (25, 26).

Data of county-level socioeconomic, demographic, and health resource were obtained from the Fujian Provincial Statistical Yearbook, an official government statistical data publishing platform (http://tjj.fujian.gov.cn/xxgk/ndsj/), including total population, rural population, gross domestic product (GDP), the number of hospital beds, *per capita* income, and *per capita* basic consumption. The carbon emission data of each county and city are obtained from the China Carbon Emission Public Database, which was derived from the DMSP/OLS and NPP/VIIRS nighttime light data provided by the National Physical Earth Data Center (NGDC) (27) and had been widely applied for carbon emission related research. (28–31)

#### 2.1.2. Variables of interest

Hospitalization rates were employed to assess the CVDs burden in each county, which was defined as the number of hospitalized cases of rural residents divided by the rural population. Carbon productivity (CP) was defined as GDP divided by carbon emissions. (31) *Per capita* gross domestic product (PGDP) was defined as GDP divided by the total population. *Per capita* income surplus ratio (PISR) was estimated by the *per capita* income and *per capita* basic consumption of rural residents in each county. Hospital beds (HB) means the number of hospital beds per 10,000 population. The proportion of low-income households (PLIH) means the proportion of low-income households among inpatients. The variable proportion of inpatients aged over 60 (PA) was adopted to reflect the aged population of the region. The above variables were calculated annually for each county during 2010–2016. The detailed measurements of variables were presented in Supplementary Table 1.

### 2.2. Statistical methods

Global Moran’s I and Local Indicators of Spatial Association (LISA) index was used to evaluate the distribution pattern of the average hospitalization rates of CVDs during study period at county level (32–34). Ordinary Least Squares (OLS) models were used to explore the global function between CVDs hospitalization rates and explanatory variables, and the variance inflation factor (VIF) was used to diagnose the collinearity of variables. Multiscale Geographically Weighted Regression (MGWR), which was extended based on Geographically Weighted Regression (GWR) and greatly improves the performance of the model MGWR though setting the bandwidth flexibly. (35–37)

The study used MGWR to explore the spatial heterogeneity of the relationship between CVDs hospitalization rates and study variables. The MGWR model expression is as follows:


$$Y_{i}=\overset{m}{\underset{j=1}{\sum }}\beta_{bwj}\left(u_{i},v_{i}\right)X_{ij}+\epsilon_{i}\;i=1,\ldots ,n$$

In the formula, $Y_{i}$ is the hospitalization rates of CVDs, X is the value of explanatory variables, β is the coefficient of explanatory variables, ε is the intercept, $u_{i},v_{i}$ denotes the coordinate of point. ${}_{bw}$ represents the bandwidth used to calibrate the j explanatoryvariable.

Geographically and Temporally Weighted Regression (GTWR) is an improved model based on the GWR model that incorporates temporal effects in the weighting matrix to measure spatial and temporal heterogeneity. (21, 22, 38) In this study, GTWR was employed to investigate the spatiotemporal heterogeneity of the relationship between CVDs hospitalization rates and explanatory variables. The GTWR model formula is as follows:


$$Y_{i}=\beta_{0}\left(u_{i},v_{i},t_{i}\right)+\underset{k}{\sum }\beta_{k}\left(u_{i},v_{i},t_{i}\right)X_{ik}+\varepsilon_{i}\;i=1,\ldots ,n$$

where $Y_{i}$ is hospitalization rates of CVDs, X is the value of explanatory variables, β is the coefficient of explanatory variables, ε is the intercept, $u_{i},v_{i},t_{i}$,denotes the space–time location.

ArcGIS 10.5 software was used to conduct Global local Moran’s I spatial autocorrelation analysis, OLS model, GTWR model, and the visualization of results. The construction of the MGWR model was done by MGWR2.2 software. Statistical significance was based on two-sided tests and was set to 5%.

## 3. Results

### 3.1. Demographic characteristics of CVDs

**Figure 1:**
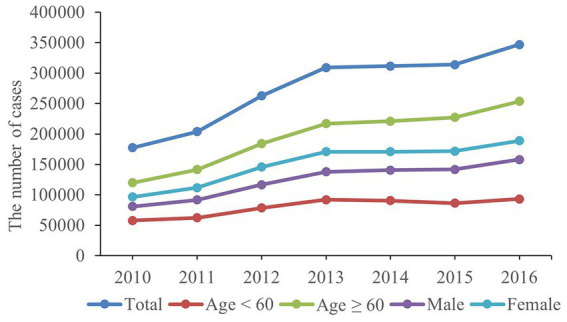
The number of rural inpatients with CVDs during 2010–2016.

**Figure 2:**
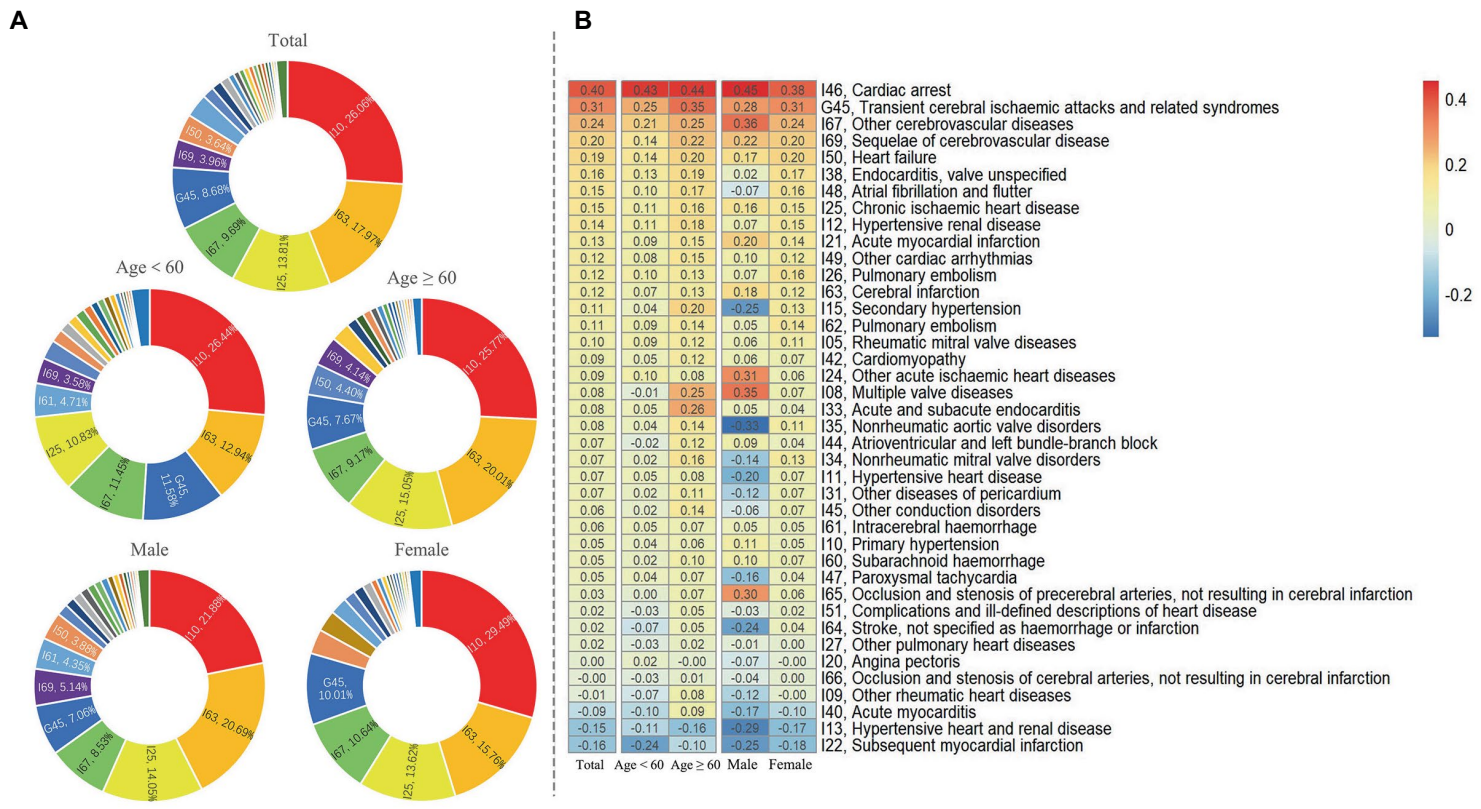
Characteristics of inpatients for subcategories diagnoses of CVDs. (**A**). The proportion of main diagnoses of CVDs. (**B**). Average annual growth rate of subcategories diagnoses of CVDs.

As shown in Figure 1 the number of rural inpatients with CVDs had increased by 95.29% from 2010 to 2016. Women had a higher number of hospitalizations compared to men (Figure 1 **and** Table 1). Hospitalizations were more frequent for individuals over 60 years of age for hypertension (70% vs. 30%), heart disease (72.6% vs. 27.4%), and cerebrovascular disease (70.3% vs. 29.7%). About 2.3% of CVD patients were from low-income households. Township (45.1%) and county-level (36.7%) hospitals were the preferred medical institutions for CVD inpatients. The highest proportion of hospitalization length was between 5 to 8 days, accounting for hypertension (49.1%), heart disease (42.1%), and cerebrovascular disease (39.4%). Figure 2A provides further details on the subgroups of CVDs. The most common hospitalized diagnoses in CVDs were essential hypertension (26.06%), cerebral infarction (17.97%), chronic ischemic heart disease (13.81%), other cerebrovascular diseases (9.69%), and transient cerebral ischemia and related syndromes (8.68%). Among patients over 60 years old, the proportion of cerebral infarction (20.01% vs. 12.94%) and chronic ischemic heart disease (15.05% vs. 10.83%) was higher, while the proportion of transient ischemic attack and associated syndromes (7.67% vs. 11.58%) was lower compared to patients under the age of 60. Women had a higher proportion of essential hypertension (29.49% vs. 21.88%) and transient ischemic attack and related syndromes (10.01% vs. 7.06%) compared to men, while the proportion of cerebral infarction was lower (15.76% vs. 20.69%). Figure 2B shows the changing trend in the number of inpatients with kinds of CVDs. The number of hospitalized patients diagnosed with cardiac arrest (39.8%), transient ischemic attack and related syndromes (31.4%), and other cerebrovascular diseases (23.5%) had the highest average percentage change. Compared with patients under 60, secondary hypertension (20.1%), multiple valve disease (26.0%), and acute and subacute endocarditis (24.9%) were the diagnoses with higher growth in among patients over 60. Besides, the average percentage change rate of acute myocardial infarction (20.5%), other acute ischemic heart disease (30.5%), and multiple valve disease was higher in male (34.8%). Figure 2B depicts the changing trend in the number of inpatients with different types of CVDs. Among them, the highest average percentage change was observed in patients diagnosed with cardiac arrest (39.8%), transient ischemic attack and related syndromes (31.4%), and other cerebrovascular diseases (23.5%). When compared to patients below 60 years, secondary hypertension (20.1%), multiple valve disease (26.0%), and acute and subacute endocarditis (24.9%) showed a higher average percentage change rate in patients over 60 years. Moreover, the average percentage change rate of acute myocardial infarction (20.5%), other acute ischemic heart disease (30.5%), and multiple valve disease was higher among males (34.8%).

**Table 1:** Characteristics of CVDs inpatients.

	Hypertension	Heart disease	cerebrovascular diseases	Total
Gender				
Male	195,115 (38.00)	250,288 (47.50)	422,226 (47.80)	867,629 (45.10)
Female	318,240 (62.00)	276,801 (52.50)	461,198 (52.20)	1,056,239 (54.90)
Age				
0 ~ 59	153,981 (30.00)	144,331 (27.40)	262,778 (29.70)	561,090 (29.10)
≥60	359,838 (70.00)	383,037 (72.60)	621,164 (70.30)	1,364,039 (70.90)
Low-income household				
Yes	11,404 (2.20)	12,951 (2.50)	20,614 (2.30)	44,969 (2.30)
No	502,415 (97.80)	514,417 (97.50)	863,328 (97.70)	1,880,160 (97.70)
Hospital level				
Township	311,257 (60.60)	186,364 (35.30)	370,524 (41.90)	868,145 (45.10)
County	149,664 (29.10)	200,690 (38.10)	355,553 (40.20)	705,907 (36.70)
Municipal	41,779 (8.10)	102,867 (19.50)	123,937 (14.00)	268,583 (14.00)
Provincial	11,118 (2.20)	37,443 (7.10)	33,923 (3.80)	82,484 (4.30)
Length of stays (days)				
0 ~ 4	108,619 (21.10)	95,525 (18.10)	136,782 (15.50)	340,926 (17.71)
5 ~ 8	252,384 (49.10)	222,097 (42.10)	348,109 (39.40)	822,590 (42.73)
9 ~ 12	98,542 (19.20)	123,372 (23.40)	196,772 (22.30)	418,686 (21.75)
≥12	54,264 (10.60)	86,372 (16.40)	202,274 (22.90)	342,910 (12.81)
Total	513,819 (100.00)	527,368 (100.00)	883,942 (100.00)	1,925,129 (100.00)

*Note: data are presented as n (%)*.

### 3.2. Geographic characteristics

**Figure 3:**
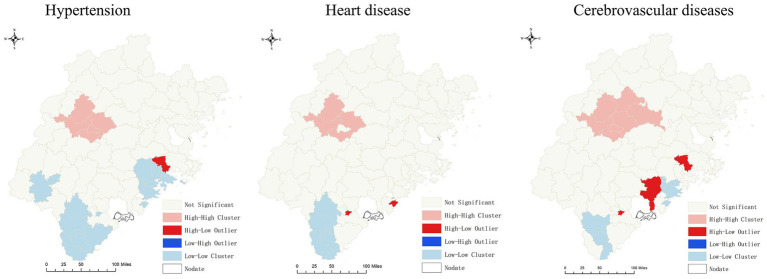
LISA agglomeration map of CVDs.

The hospitalization rates for hypertension, heart disease, and cerebrovascular disease among rural residents from 2010 to 2016 are presented in Supplementary Figure 1, with all three showing an increasing trend. Notably, the hospitalization rates for all types of CVDs increased to a greater extent in central and midwestern counties. Additionally, Table 2 shows that the Moran’s I index for all three types of CVDs hospitalization rates was statistically significant (*p* < 0.05), indicating signifificant spatial autocorrelation in the study area. Furthermore, the LISA agglomeration map revealed a high-high cluster pattern for CVDs hospitalization rates in central and midwestern counties, including Meilie, Sanyuan, Mingxi, Jiangle, and Shaxian, while the southern region exhibited a low-low cluster pattern, as illustrated in Figure 3. These findings suggest the need for further investigation into the hospitalization phenomenon of CVDs in central and midwestern counties.

**Table 2:** Moran’s I Index of Hospitalization rates for CVDs.

Year	Hypertension			Heart disease			Cerebrovascular disease		
	I	*Z*	*P*	I	*Z*	*P*	I	*Z*	*P*
2010	0.548111	7.101323	<0.001	0.269025	3.239312	0.001	0.350378	4.623761	<0.001
2011	0.503750	6.511521	<0.001	0.224342	2.727595	0.006	0.391295	4.954839	<0.001
2012	0.527301	6.494935	<0.001	0.161347	1.997368	0.004	0.351631	4.412138	<0.001
2013	0.393643	5.772436	<0.001	0.19912	2.488498	0.012	0.399185	5.023373	<0.001
2014	0.302253	5.649183	<0.001	0.180847	2.805158	0.050	0.531419	6.956130	<0.001
2015	0.212676	4.483808	<0.001	0.180508	3.381287	<0.001	0.47621	6.257808	<0.001
2016	0.178785	3.518335	<0.001	0.160382	2.876363	0.004	0.390991	5.085847	<0.001

#### 3.2.1. Influencing factors of CVDs

The OLS model analysis results are presented in Table 3, revealing that CVDs hospitalization rates were positively correlated with PGDP, HB, PA, and PLIH (*β* > 0, *p* < 0.05), but negatively correlated with CP (*β* < 0, p < 0.05) at the county level. VIF of each variable ranged from 1 to 1.45, indicating no significant multi-collinearity among variables. To explore the spatiotemporal heterogeneity of CVDs and variable relationships, the limitations of the OLS model in explaining spatial heterogeneity of associations were addressed by using the more robust MGWR and GTWR models. Table 4 and Table 5 display the results of both MGWR and GTWR models, which demonstrated similar outcomes, although each model has its strengths. The MGWR model displayed greater flexibility in adjusting variable bandwidth, resulting in a lower Akaike Information Criterion corrected (AICc) than the GTWR model. Conversely, the GTWR model had a higher adjusted R^2^ due to better measurement of the spatiotemporal heterogeneity of the association.

**Table 3:** The correlation between CVDs hospitalization rate and factors by OLS model.

Diseases	Variables	β (95%*CI*)	*P*	VIF	Adjusted R^2^
Hypertension	Intercept	−186.982(−252.045, −121.918)	<0.001		0.265
	PGDP	10.835 (6.947,14.724)	<0.001	1.435	
	CP	−37.908(−57.441, −18.375)	<0.001	1.272	
	PISR	0.500(−0.300,1.300)	0.220	1.076	
	PLIH	2.363(−0.858,5.584)	0.150	1.089	
	PA	1.981 (1.216,2.746)	<0.001	1.052	
	HB	1.988 (1.541,2.435)	<0.001	1.362	
Heart diseases	Intercept	−77.445(−109.519, −45.371)	<0.001		0.353
	PGDP	8.502 (6.445,10.558)	<0.001	1.426	
	CP	−8.964(−19.452,1.524)	0.094	1.303	
	PISR	−0.04(−0.464,0.385)	0.855	1.075	
	PLIH	3.848 (2.350,5.346)	<0.001	1.076	
	PA	0.859 (0.465,1.252)	<0.001	1.122	
	HB	0.808 (0.574,1.043)	<0.001	1.33	
Cerebrovascular diseases	Intercept	−92.266(−180.641, −3.89)	0.041		0.350
	PGDP	18.215 (13.999,22.431)	<0.001	1.49	
	CP	−21.693(−42.393, −0.993)	0.040	1.263	
	PISR	−0.846(−1.694,0.002)	0.051	1.069	
	PLIH	3.962 (0.539,7.385)	0.023	1.100	
	PA	0.931(−0.269,2.131)	0.128	1.097	
	HB	1.802 (1.33,2.274)	<0.001	1.343	

β*: represents the strength and type of relationship between each explanatory variable and the dependent variable. Probability (P): Coefficient is statistically significant (P < 0.05). Variance inflation factor (VIF): large VIF values (>7.5) indicate redundancy among explanatory variables. Adjusted R^2^: the fraction of the variance in the data that is explained by the model*.

**Table 4:** Summary of MGWR model result.

Diseases	Variables	β			Adjusted *R*^2^	AICc
		mean	min	max		
Hypertension	Intercept	−0.282	−0.819	0.364	0.874	516.992
	PGDP	0.193	−0.537	1.259		
	CP	−0.208	−1.072	0.058		
	PISR	0.077	−0.108	0.335		
	PLIH	0.052	−0.161	0.290		
	PA	0.246	−0.262	1.364		
	HB	0.168	−0.216	0.805		
Heart diseases	Intercept	−0.120	−0.755	0.300	0.825	664.748
	PGDP	0.339	−0.209	1.232		
	CP	−0.026	−0.304	0.292		
	PISR	0.030	−0.001	0.075		
	PLIH	0.181	−0.202	1.183		
	PA	0.320	−0.565	1.288		
	HB	0.112	−0.002	0.275		
Cerebrovascular diseases	Intercept	0.025	−0.802	1.777	0.884	492.024
	PGDP	0.617	0.051	1.997		
	CP	−0.003	−0.492	0.324		
	PISR	−0.067	−0.084	−0.060		
	PLIH	0.123	−0.317	1.622		
	PA	0.071	−0.504	0.468		
	HB	−0.120	−0.772	0.654		

β*: represents the strength and type of relationship between each explanatory variable and the dependent variable. Adjusted R^2^: the fraction of the variance in the data that is explained by the model. Akaike Information Criterion (AICc): A lower AICc reflects a better fit given a penalty for increasing the number of parameters*.

**Table 5:** Summary of GTWR model result.

Diseases	Variable	β			Adjusted R^2^	AICc
		mean	min	max		
Hypertension	Intercept	−113.836	−1373.785	197.439	0.893	4918.090
	PGDP	5.751	−31.164	76.426		
	CP	−32.709	−182.440	40.486		
	PISR	0.329	−3.399	10.295		
	PLIH	2.538	−16.776	22.288		
	PA	1.593	−2.220	10.871		
	HB	1.061	−0.339	5.758		
Heart diseases	Intercept	−80.670	−1044.922	209.687	0.885	4462.080
	PGDP	4.957	−11.335	49.436		
	CP	−5.994	−62.967	41.646		
	PISR	−0.021	−2.893	6.991		
	PLIH	3.866	−6.498	22.489		
	PA	1.172	−2.484	10.532		
	HB	0.508	−0.570	2.258		
Cerebrovascular diseases	Intercept	−37.102	−1069.043	380.494	0.907	5036.690
	PGDP	16.141	−30.809	113.255		
	CP	−16.918	−238.517	165.162		
	PISR	−1.182	−10.703	4.485		
	PLIH	6.569	−15.390	51.302		
	PA	0.905	−5.639	11.207		
	HB	0.420	−4.559	6.672		

β *: represents the strength and type of relationship between each explanatory variable and the dependent variable. Adjusted R^2^: the fraction of the variance in the data that is explained by the model. Akaike Information Criterion (AICc): A lower AICc reflects a better fit given a penalty for increasing the number of parameters*.

**Figure 4:**
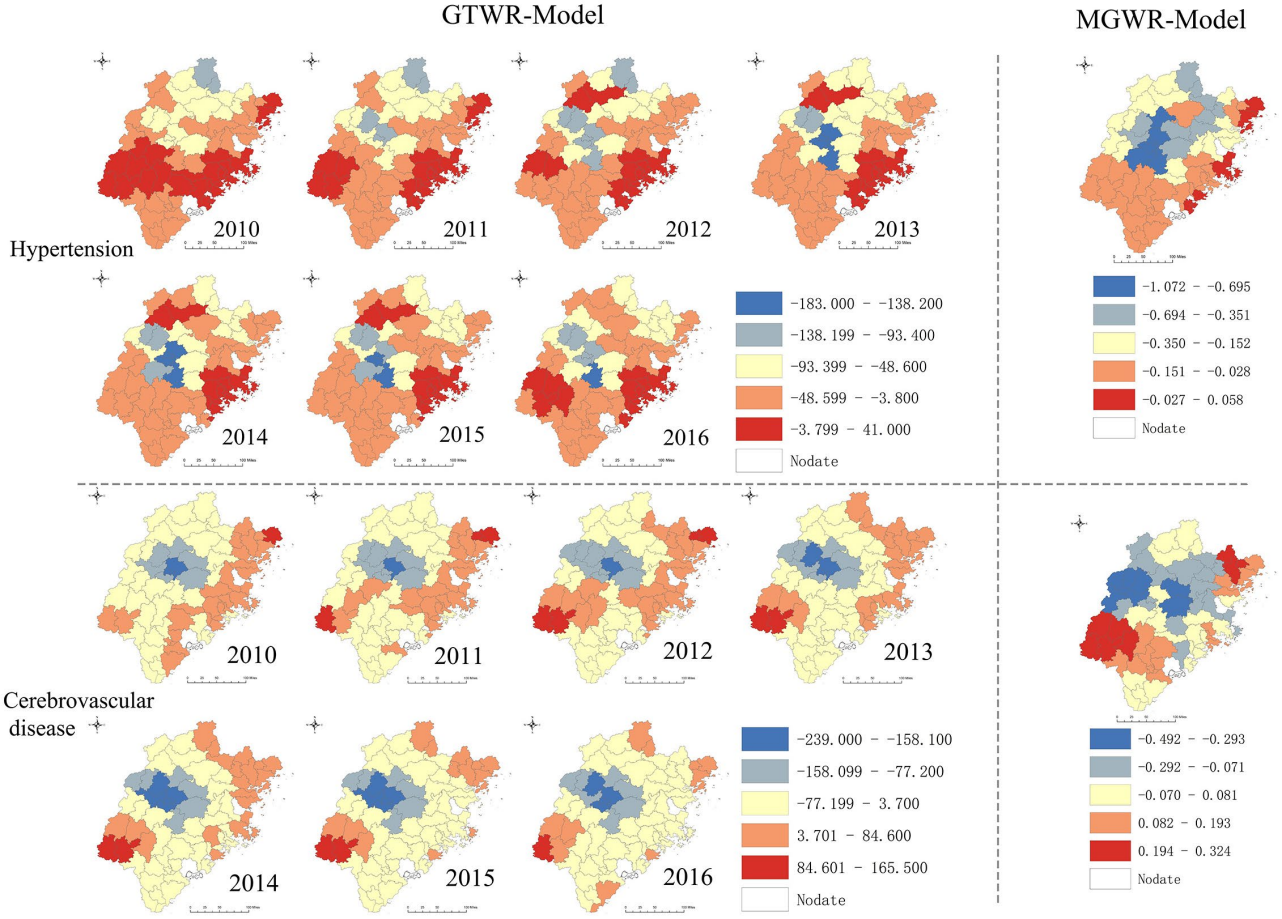
Spatiotemporal heterogeneity of coefficient for CP.

**Figure 5:**
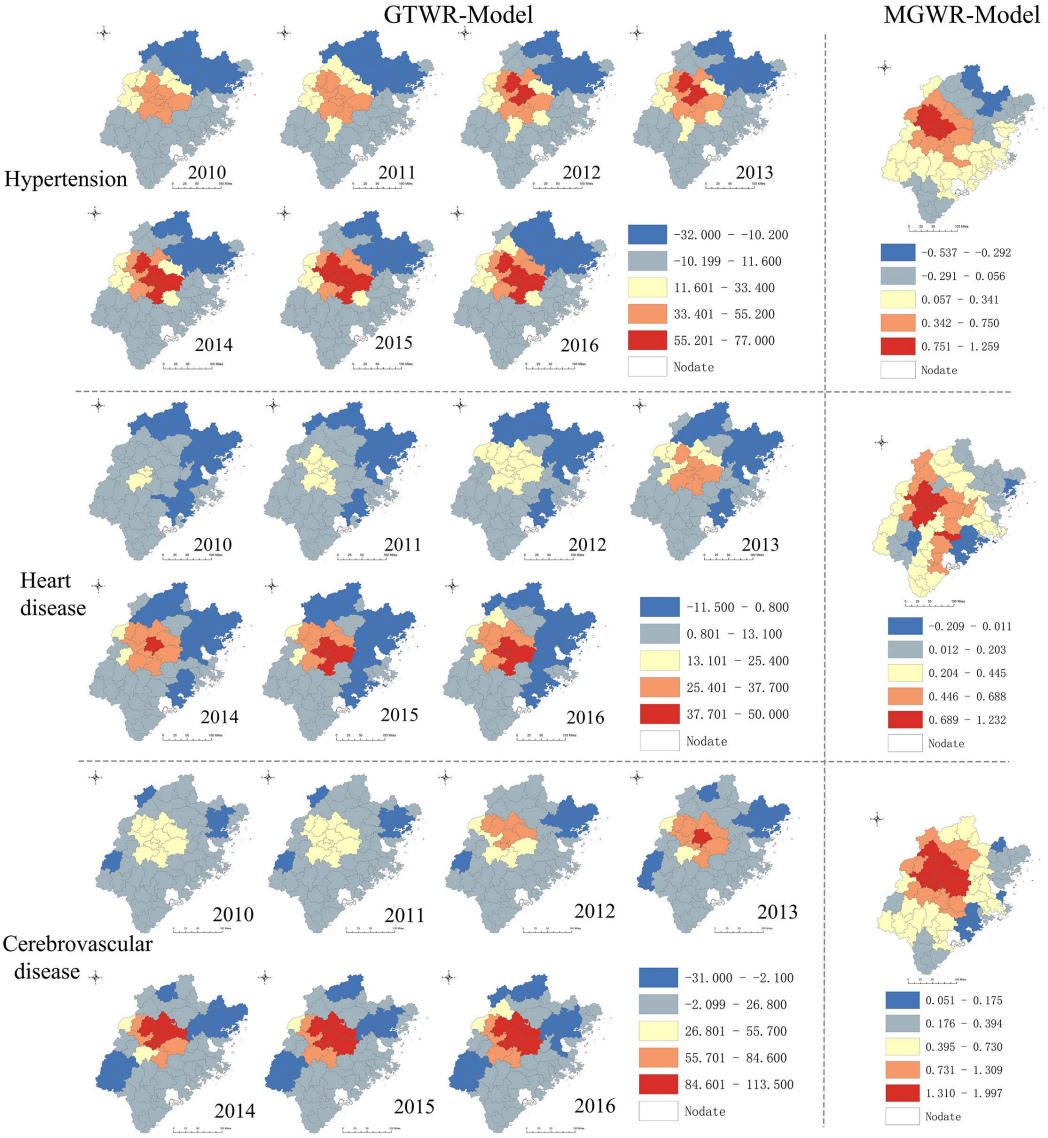
Spatiotemporal heterogeneity of coefficient for PGDP.

Figure 4 shows the relationship between CP and hospitalization rates of hypertension and cerebrovascular disease. Consistent with the findings in Tables 3–5, there was a negative correlation between CP and hospitalization rates, particularly in central and midwestern counties where the negative correlation effect was more pronounced. The trend towards negative correlation was observed to be increasing from 2010 to 2016. The spatiotemporal distribution characteristics of the coefficients for PGDP and PLIH were analyzed and compared in Figure 5 and Supplementary Figure 2. The results showed that there were considerable positive correlations between hospitalization rate CVDs and PGDP and PLIH in most counties during the study period, with the correlation being stronger in central and midwestern counties. The trend of this positive correlation also increased during the study period. Supplementary Figure 3 displayed the spatiotemporal distribution characteristics of the coefficients for PA, revealing a stronger and increased positive correlation between PA and hypertension in the central and northeastern counties. Similar findings were observed for heart disease in the southwest and northeast regions of the study area. Additionally, Supplementary Figure 4 showed the spatiotemporal distribution characteristics of the coefficients for HB, indicating that the positive correlation between HB and CVDs was stronger in the northern part of the study area, with slight temporal heterogeneity of coefficients.

## 4. Discussion

This study has comprehensively explored the characteristics of rural inpatients with CVDs in southeast China by sex, age, and spatiotemporal distribution. We observed that the number of rural inpatients with CVDs had been rising dramatically. Essential hypertension, cerebral infarction, and chronic ischemic heart disease were the leading causes of hospitalization. In addition, to the best of our knowledge, this is the first spatial analysis study to assess the association between carbon productivity (CP) and health outcomes. We observed that CVDs hospitalization rates were negatively associated with CP, and positively associated with *per capita* GDP (PGDP) and hospital beds per 10,000 population (HB) in most counties.

We observed that the number of rural inpatients with CVDs had increased by 95.29% from 2010 to 2016. The majority of inpatients were elderly. Cardiac arrest, transient ischemic attack and related syndromes had the highest average percentage change rate. The fast-growing inpatients with CVDs was possibly related to the development of Chinese society and the intensification of aging problems, changes in people’s lifestyles, and medical and health care. Consistently, relevant studies had pointed out that with the aging process of Chinese society in the 21st century, the prevalence and incidence of age-related diseases have risen sharply. (39) Higher age was an independent risk factor for CVDs. Among age-related diseases, CVDs represented by heart disease and cerebral infarction are the main causes of death. (40, 41) In addition, after China’s long-term construction of the medical system, more rural residents have access to medical services, which may also be the reason for the increase of CVDs inpatients. (24, 42)

In our study, the number of female inpatients for CVDs was higher than male. Previous studies used to consider CVDs as a male-predominant disease. (43) However, CVDs were also the leading cause of death and loss of disability-adjusted life years for women worldwide. (44) This phenomenon may be attributed to insufficient attention to the prevention of CVDs in women. Studies have shown that in the United States, compared with men, women with CVDs were more likely to be underdiagnosed and not receive preventive care or appropriate treatment. (45) In China, women place less emphasis on primary and secondary prevention of CVDs than men, who may experience heart disease in different ways, and lack of awareness of it, which had been revealed to lead to adverse outcomes. (46, 47) Therefore, it is necessary to redefine the meaning of women’s health to cover the entire life cycle, with more emphasis on the prevention of CVDs and other non-communicable diseases.

We observed that the hospitalization rates for rural inpatients with CVDs were not discretely distributed in southeast China, but exhibit obvious aggregated in central and midwestern counties, such as Meilie, Sanyuan, Mingxi, Jiangle, and Shaxian. Consistently, the distribution of CVDs burden varies greatly different in regions of China. Coastal provinces with higher economic development have a lower CVDs burden than inland regions, and northern regions with more severe air pollution are higher than southern regions. (8, 48) Therefore, to achieve higher health policy benefits, it is suggested that the government should formulate targeted health policies to reduce the CVDs burden based on the multi-dimensional characteristics of each county such as economy, health resources, population, and CVDs burden.

We found that higher CP was negatively associated with the hospitalization rate of CVDs, while PGDP was positively associated with them. These correlations were stronger in central and midwestern counties. These results suggest that low-carbon development is beneficial in reducing the burden of CVDs. The abusive and inefficient use of non-renewable energy may improve the regional economy in the short term, but the cost is the emission of a large amount of greenhouse gases, resulting in air pollution and destruction of soil and water quality, and ultimately lead to an increase in the burden of CVDs. (8–10, 12) In study area, the central and midwestern counties were economically underdeveloped regions, there may exist a large number of inefficient and highly polluting industries and technologies, resulting in massive carbon emissions and environmental pollution. In order to mitigate the disease burden of CVDs, it is imperative for the government to promote a balance between economic development and environmental protection. We recommend implementing policies such as carbon trading, (49) promoting renewable energy, (50) rationally adjusting the industrial structure, (51) and reducing reliance on fossil fuels. Such measures can not only help reduce carbon emissions, but also have the potential to improve public health, promote economic development and reduce the incidence of CVDs.

We observed that there was a stronger positive association between proportion of low-income households (PLIH) and hospitalization rate of heart diseases and cerebrovascular diseases in central and the western parts. Consistently, research had shown that poverty is a risk factor for CVDs. (52, 53) Low-income households were more likely to be unable to afford primary and secondary prevention services for CVDs, leading to disease progression and ultimately hospitalization. Therefore, in central and the western counties need to pay more attention to the problem of the gap between the rich and the poor. On the other hand, it is worth considering increasing the coverage of public medical insurance and the reimbursement ratio of medical expenses for the poor. (54)

We found that HB had a positive function on the hospitalization rate of CVDs, especially in the northern and central counties. The results indicate a significant imbalance in the distribution of medical resources in the southeast rural China. Consistently, Previous studies have suggested that the distribution of health resources in Fujian mainly concentrated in economically developed regions like Fuzhou, Xiamen, Quanzhou, and other coastal areas, while health resources are scarce in the northern. (23) With the Health-Care Reforms in China, rural residents can receive medical services more conveniently, (24, 42) which promotes an increase in the number of hospitalizations. However, the uneven distribution of medical resources is still an issue. (55, 56) In some counties, the increase in medical and health resources may not be sufficient to meet the health needs of the population, leading to difficulties and delays in seeking medical treatment. In contrast, other areas may have sufficient medical and health resources, and further increasing resources may not significantly increase the number of patients seeking medical treatment. Therefore, in order to better meet the health needs of the population, China’s healthcare system still needs further improvement, especially in underdeveloped inland areas. More rational allocation of medical and health resources is a key step in achieving this goal.

Our study has several limitations that should be noted. Firstly, medical insurance policies can vary between counties based on their financial situation, potentially affecting the hospitalization rates of rural residents with CVDs. Second, the NRCMS database only records the main diagnoses of inpatients, while many patients often have multiple diseases simultaneously, which may lead to an underestimation of the number of hospitalizations due to CVDs. Thirdly, the NRCMS is a healthcare insurance policy for rural China, and all study participants are exclusively rural residents. Therefore, caution should be exercised when extrapolating the study’s results to urban areas due to significant differences in demographic and environmental characteristics. Finally, our study is subject to the risk of ecological fallacies as it was conducted at the county level. To mitigate this limitation, future research using individual-level data would be beneficial to validate our findings and assess their generalizability.

## 5. Conclusion

The number of rural inpatients with CVDs had been rising dramatically. The main groups affected were elderly and women, with essential hypertension, cerebral infarction, and chronic ischemic heart disease were the leading causes of hospitalization. In addition, the CVDs hospitalization rates were negatively associated with CP, and positively associated with PGDP and HB in most counties. The association between CVDs hospitalization rates and CP and *per capita* GDP was stronger in central and midwestern counties, while the association with HB was stronger in northern counties. Based on our findings, we recommend taking the following measures to address the increasing inpatients with CVDs. First, conducting more CVDs prevention work in rural areas, particularly in central and midwestern counties. Second, balancing social development and environmental protection by regard CP as metric for evaluating the environmental health. Finally, allocating medical resources more rationally, especially in northern counties.

## Data availability statement

The original contributions presented in the study are included in the article/Supplementary material, further inquiries can be directed to the corresponding author/s.

## Author contributions

ZH, JJ and XT conceptualized and led the study. XT and ZZ designed the study. Data collection was done by XT and ZZ. XT, ZZ and ZR contributed to data analysis. The first draft of the article was written by XT. ZZ, ZR, HF, and XH reviewed the manuscript and provided critical inputs. All authors contributed to the article and approved the submitted version.

## Funding

This work was supported by the Provincial Natural Science Foundation of Fujian Province (No. 2021J01728), the Scientific and Innovation Capital Projects of Fujian Province (No. 2022R0088), the Fujian Provincial Health Science and Technology Plan Project (No. 2019-RK-5), and the Fujian Provincial Medical and Health System Reform Research Association Health Policy Innovation Research Project China (No. 2022B07).

## Conflict of interest

The authors declare that the research was conducted in the absence of any commercial or financial relationships that could be construed as a potential conflict of interest.

## Publisher’s note

All claims expressed in this article are solely those of the authors and do not necessarily represent those of their affiliated organizations, or those of the publisher, the editors and the reviewers. Any product that may be evaluated in this article, or claim that may be made by its manufacturer, is not guaranteed or endorsed by the publisher.
