# Supplementary figures and images for: A spatiotemporal analysis of the association between carbon productivity, socioeconomics, medical resources and cardiovascular diseases in southeast rural China
Source: Front Public Health. 2023 Jul 6;11:1079702. doi: 10.3389/fpubh.2023.1079702 (PMC10359911; doi:10.3389/fpubh.2023.1079702)

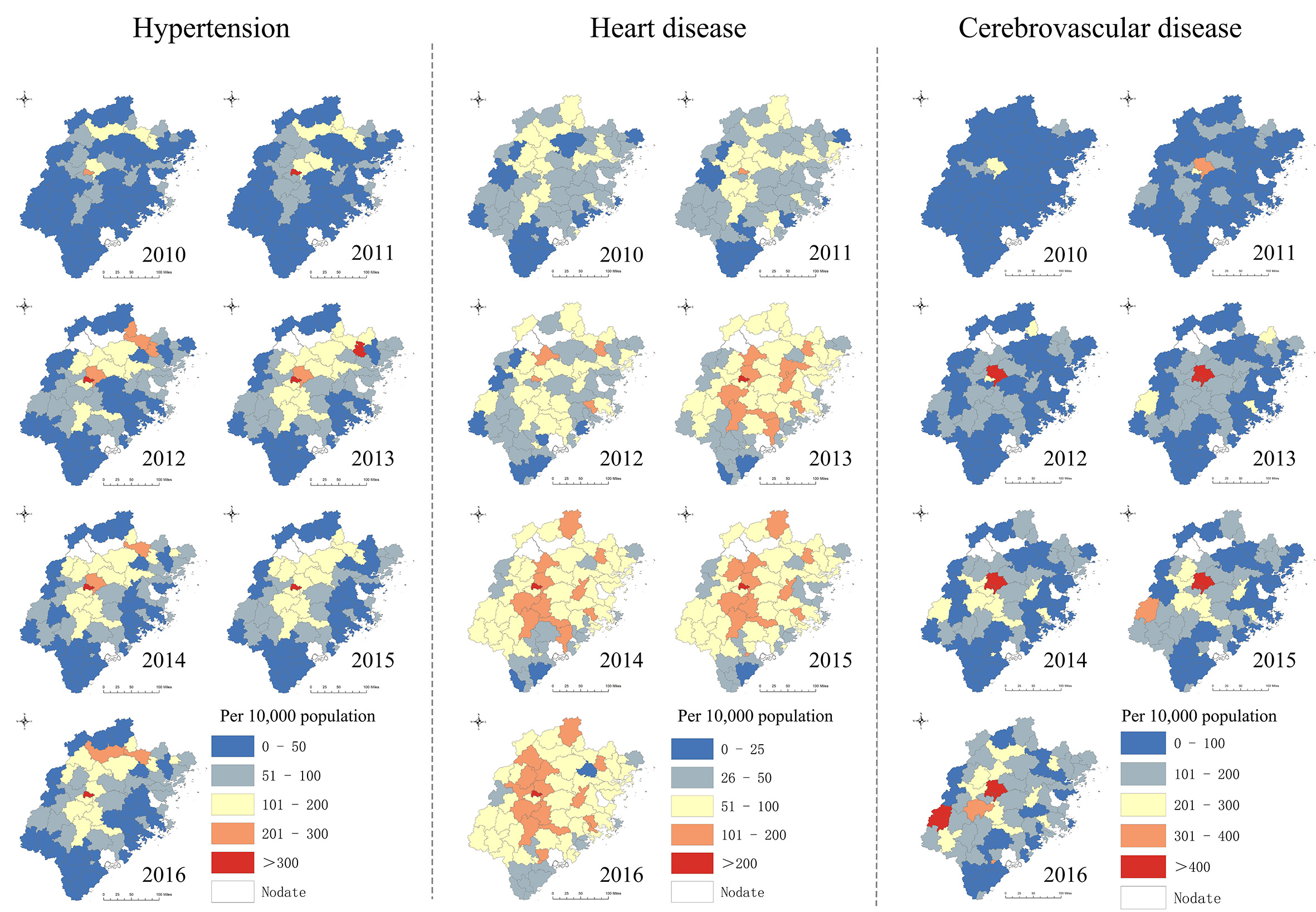

Supplement: Supplementary file 1 [file Image_1.JPEG]

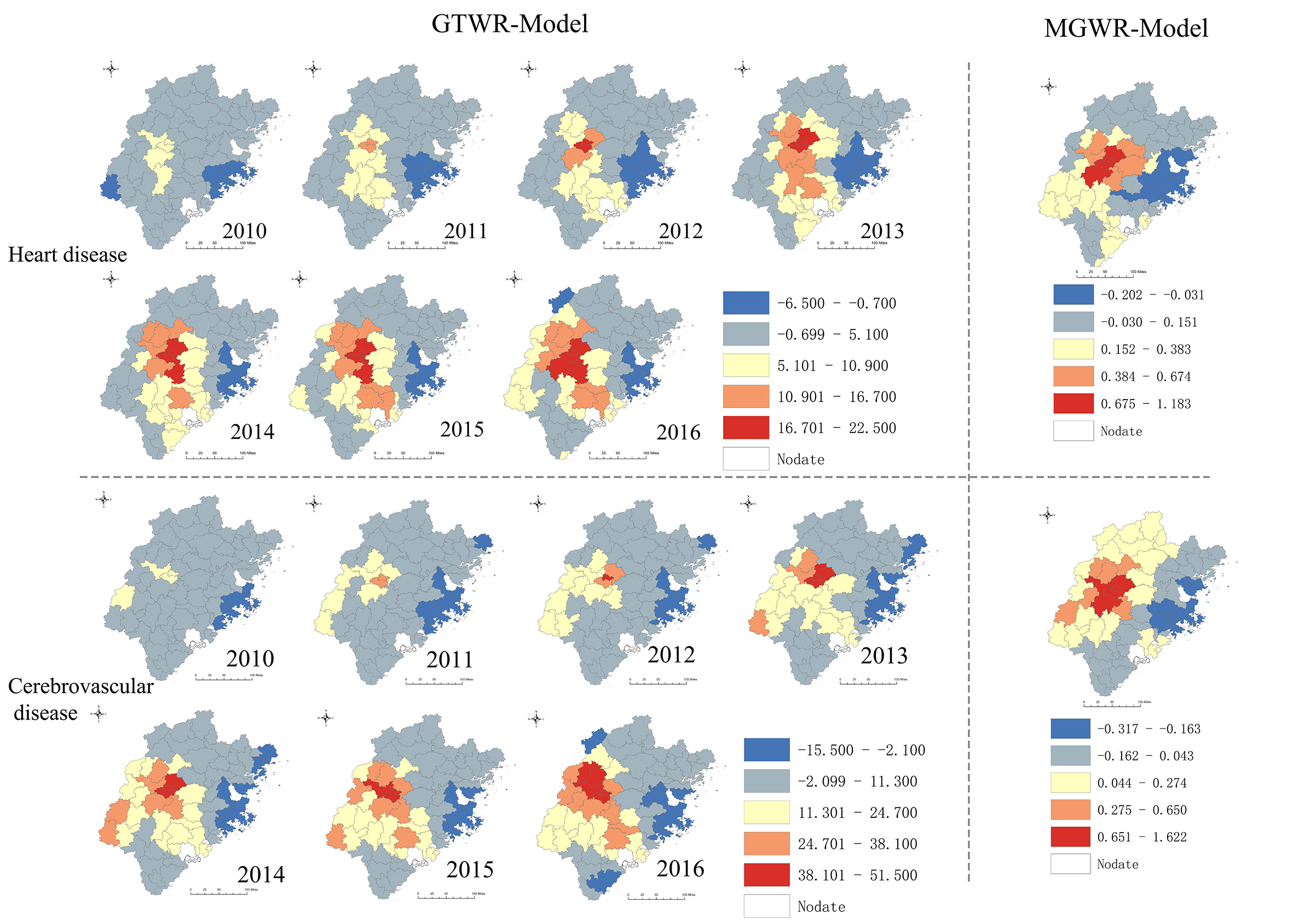

Supplement: Supplementary file 2 [file Image_2.JPEG]

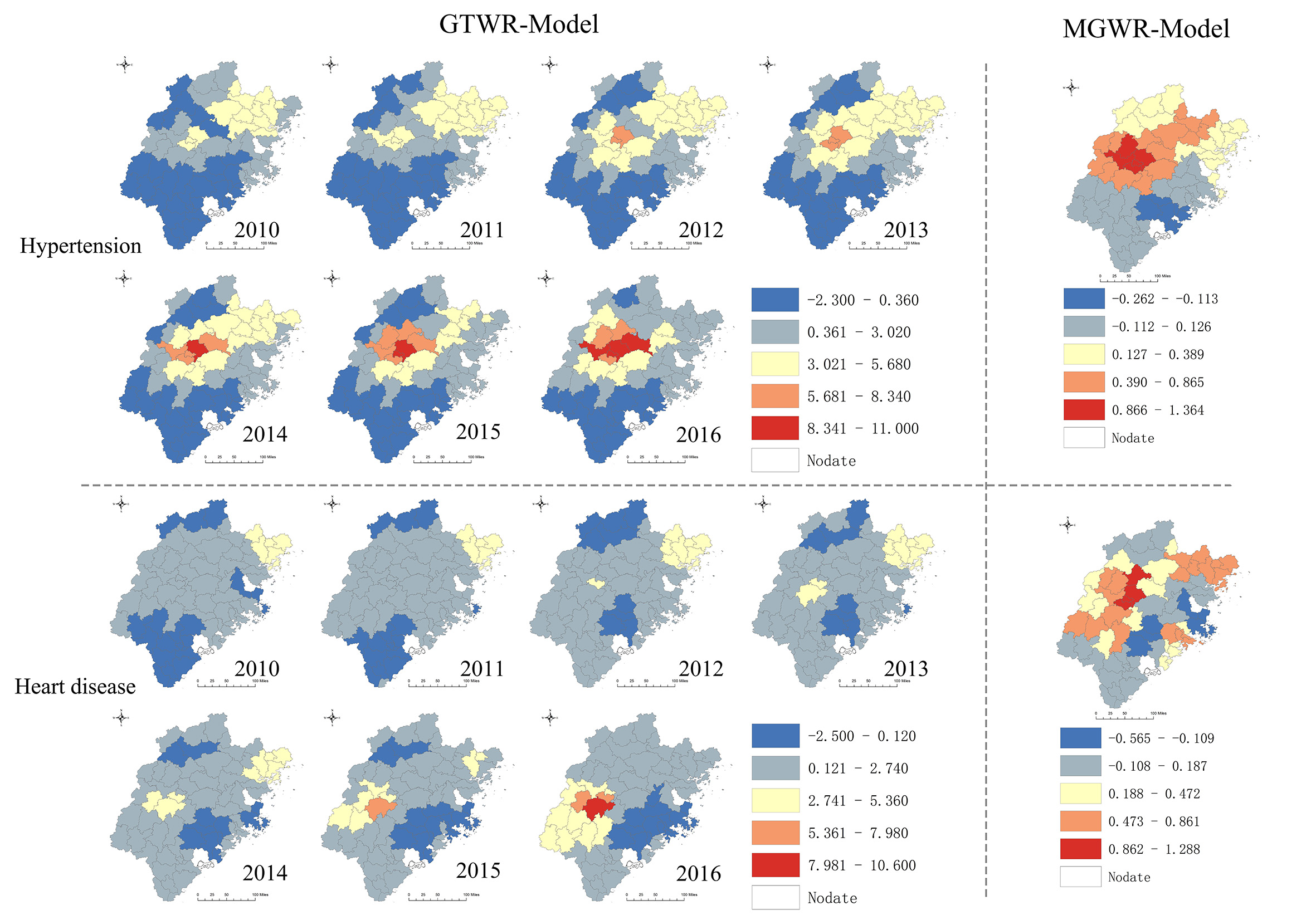

Supplement: Supplementary file 3 [file Image_3.JPEG]

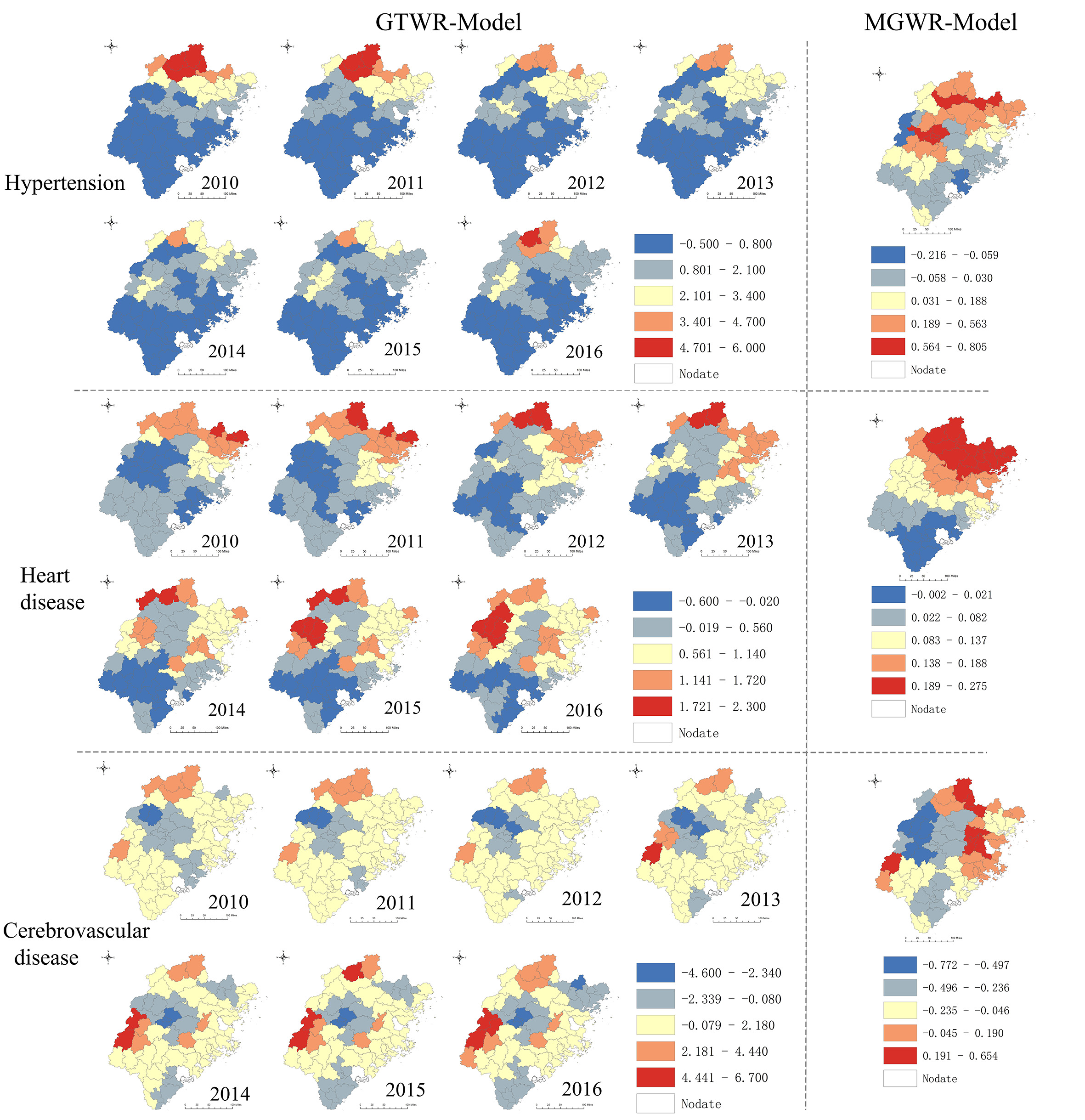

Supplement: Supplementary file 4 [file Image_4.JPEG]
