# Supplementary material for: A spatiotemporal analysis of the association between carbon productivity, socioeconomics, medical resources and cardiovascular diseases in southeast rural China
Source: Front Public Health. 2023 Jul 6;11:1079702. doi: 10.3389/fpubh.2023.1079702 (PMC10359911; doi:10.3389/fpubh.2023.1079702)
Supplement: Supplementary file 5 [file Table_1.DOCX]

Supplementary Material

# Supplementary Data

Supplementary Material should be uploaded separately on submission. Please include any supplementary data, figures and/or tables. All supplementary files are deposited to FigShare for permanent storage and receive a DOI.

Supplementary material is not typeset so please ensure that all information is clearly presented, the appropriate caption is included in the file and not in the manuscript, and that the style conforms to the rest of the article. To avoid discrepancies between the published article and the supplementary material, please do not add the title, author list, affiliations or correspondence in the supplementary files.

# Supplementary Figures and Tables

**Supplementary Table 1.** The detailed measurements of variables

| Variables | Measurements | Unit | Database |
| --- | --- | --- | --- |
| Per capita gross domestic product (PGDP) | gross domestic product / total population | 10,000Yuan/person | Statistical Yearbook |
| Carbon productivity (CP) | gross domestic product / carbon emissions | 10,000Yuan /ton | Statistical Yearbook, China Carbon Emission Database |
| Per capita income surplus ratio (PISR) | (per capita income - per capita basic consumption)/per capita income | % | Statistical Yearbook |
| Hospital beds (HB) | number of hospital beds/total population | n/10,000 population | Statistical Yearbook |
| Proportion of low-income households (PLIH) | number of low-income households/ number of patients | % | NRCMS |
| Proportion of inpatients aged over 60 (PA) | number of patients over 60 years old/number of patients | % | NRCMS |
