# Supplementary material for: A spatiotemporal analysis of the association between carbon productivity, socioeconomics, medical resources and cardiovascular diseases in southeast rural China
Source: Front Public Health. 2023 Jul 6;11:1079702. doi: 10.3389/fpubh.2023.1079702 (PMC10359911; doi:10.3389/fpubh.2023.1079702)
Supplement: Supplementary file 6 [file Table_2.DOCX]

# Supplementary Figures

#
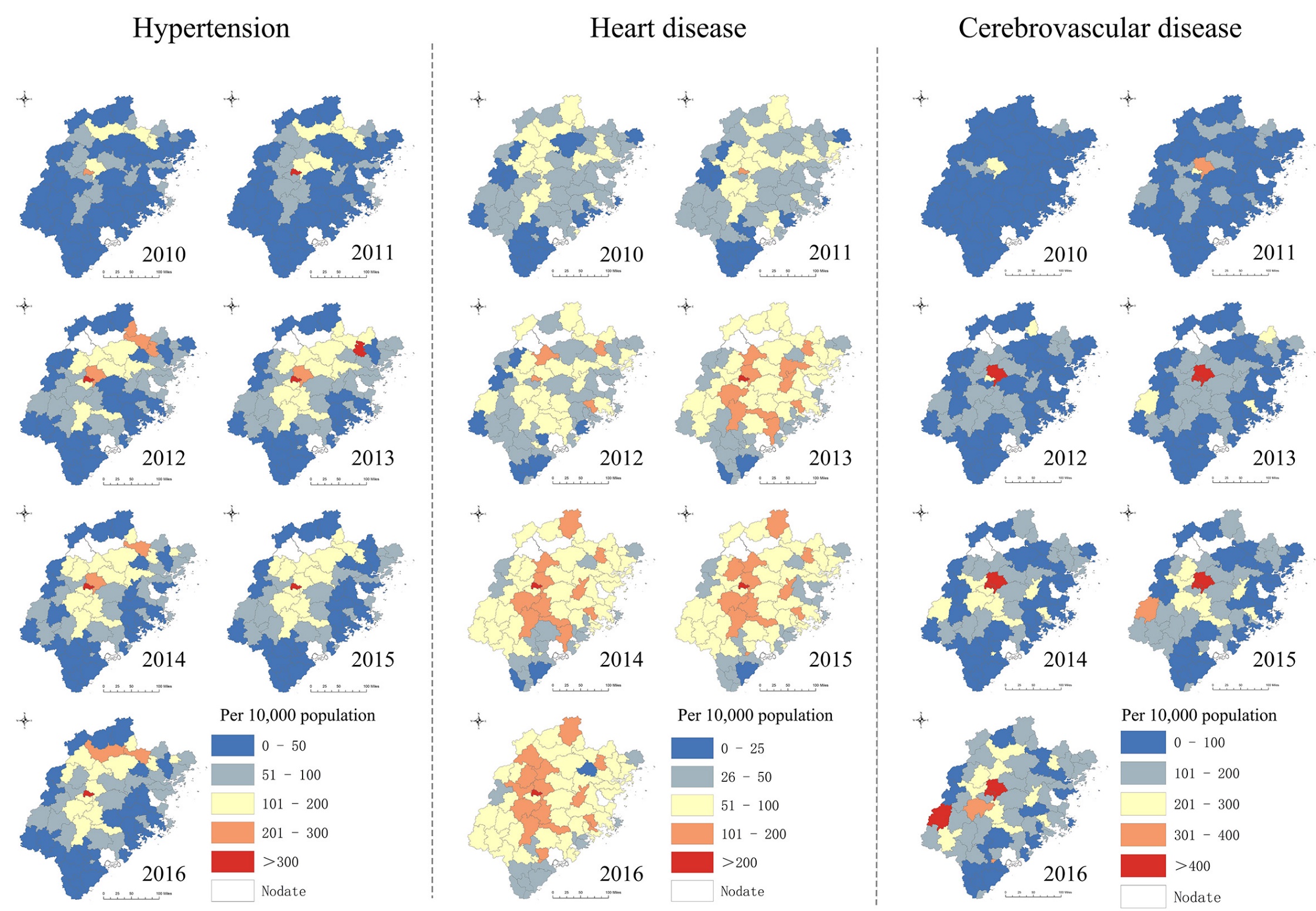


**Supplementary Figure 1 Hospitalization rates for CVDs among rural residents, 2010 to 2016.**


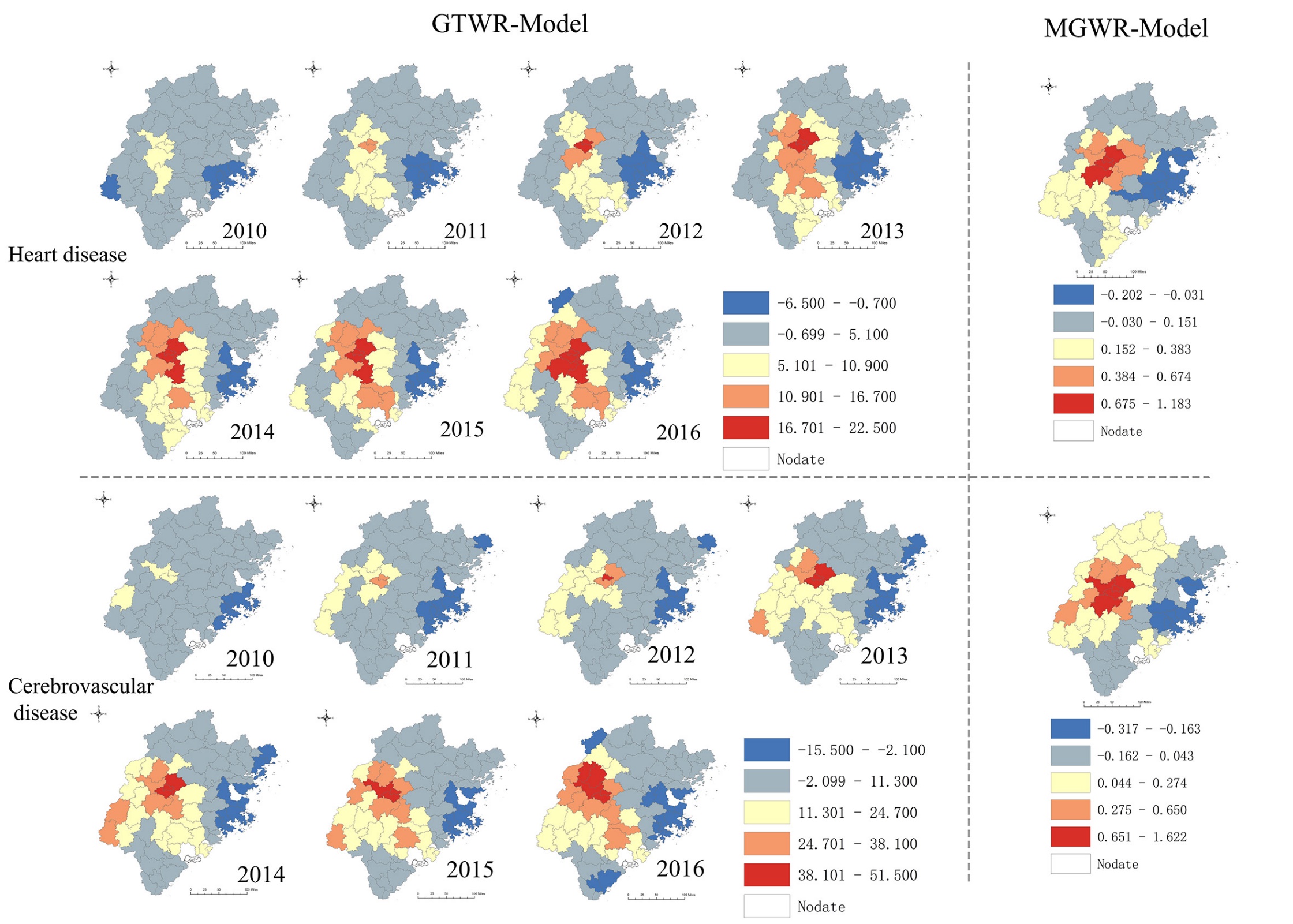


**Supplementary Figure2** Spatiotemporal heterogeneity of coefficient for PLIH


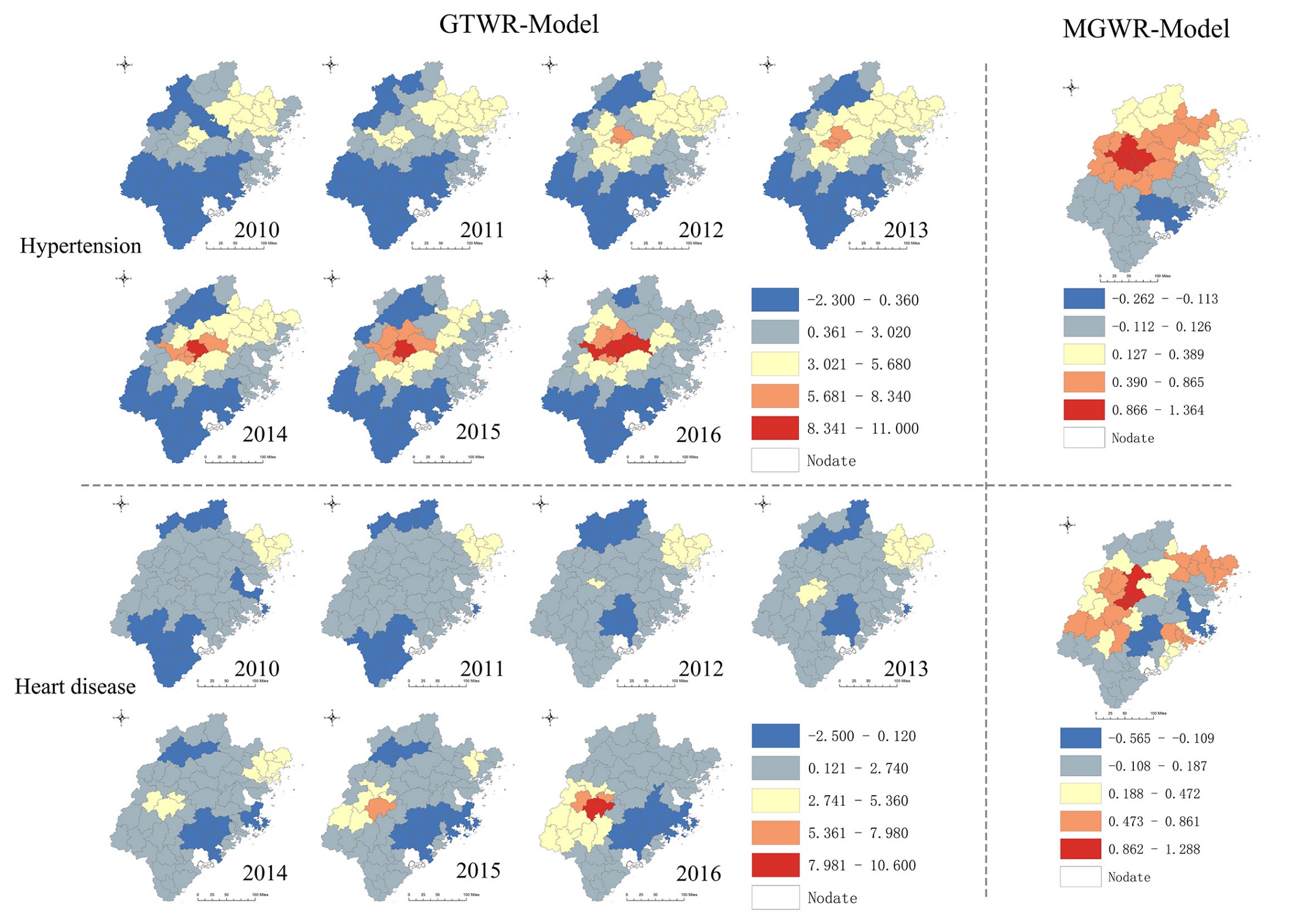


**Supplementary Fig.3** Spatiotemporal heterogeneity of coefficient for PA


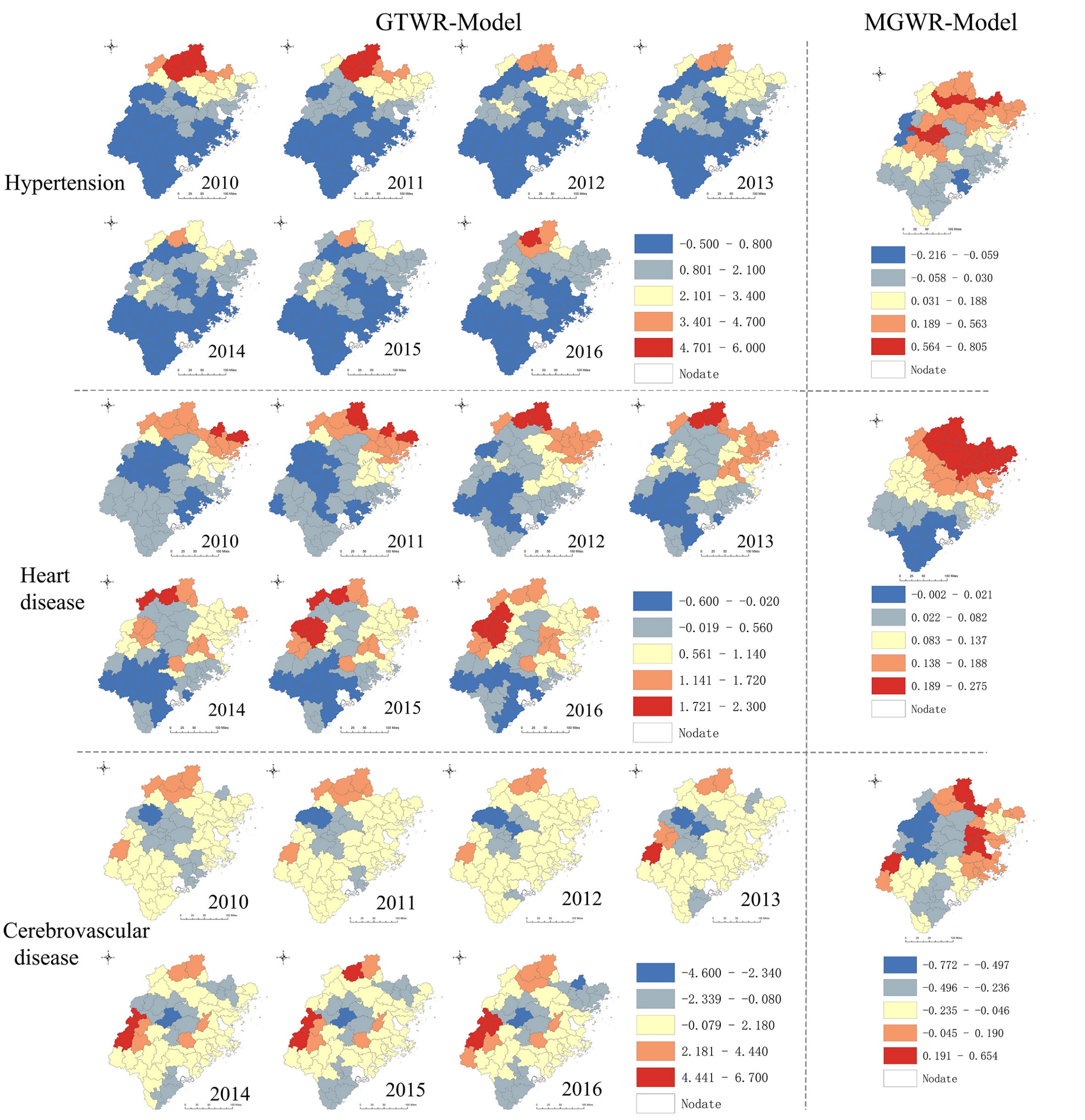


**Supplementary Fig.4 Spatiotemporal heterogeneity of coefficient for HB**
